# A whole cell fluorescence quenching-based approach for the investigation of polyethyleneimine functionalized silver nanoparticles interaction with *Candida albicans*

**DOI:** 10.3389/fmicb.2023.1131122

**Published:** 2023-02-28

**Authors:** Atul Kumar Tiwari, Munesh Kumar Gupta, Roger J. Narayan, Prem C. Pandey

**Affiliations:** ^1^Department of Chemistry, Indian Institute of Technology (BHU), Varanasi, Uttar Pradesh, India; ^2^Mycology Laboratory, Department of Microbiology, Institute of Medical Sciences, Banaras Hindu University, Varanasi, Uttar Pradesh, India; ^3^Joint Department of Biomedical Engineering, University of North Carolina and North Carolina State University, Chapel Hill, NC, United States

**Keywords:** autofluorescence, fluorescence quenching, silver nanoparticles, intrinsic fluorescence, surface expressed proteins

## Abstract

The antimicrobial activity of metal nanoparticles can be considered a two-step process. In the first step, nanoparticles interact with the cell surface; the second step involves the implementation of the microbicidal processes. Silver nanoparticles have been widely explored for their antimicrobial activity against many pathogens. The interaction dynamics of functionalized silver nanoparticles at the biological interface must be better understood to develop surface-tuned biocompatible nanomaterial-containing formulations with selective antimicrobial activity. Herein, this study used the intrinsic fluorescence of whole *C. albicans* cells as a molecular probe to understand the cell surface interaction dynamics of polyethyleneimine-functionalized silver nanoparticles and antifungal mechanism of the same. The results demonstrated that synthesized PEI-f-Ag-NPs were ~ 5.6 ± 1.2 nm in size and exhibited a crystalline structure. Furthermore, the recorded zeta potential (+18.2 mV) was associated with the stability of NPS and shown a strong electrostatic interaction tendency between the negatively charged cell surface. Thus, rapid killing kinetics was observed, with a remarkably low MIC value of 5 μg/mL. PEI-f-Ag-NPs quenched the intrinsic fluorescence of *C. albicans* cells with increasing incubation time and concentration and have shown saturation effect within 120 min. The calculated binding constant (Kb = 1 × 10^5^ M^−1^, *n* = 1.01) indicated strong binding tendency of PEI-f-Ag-NPs with *C. albicans* surface. It should also be noted that the silver nanoparticles interacted more selectively with the tyrosine-rich proteins in the fungal cell. However, calcofluor white fluorescence quenching showed non-specific binding on the cell surface. Thus, the antifungal mechanisms of PEI-f-Ag-NPs were observed as reactive oxygen species (ROS) overproduction and cell wall pit formation. This study demonstrated the utility of fluorescence spectroscopy for qualitative analysis of polyethyleneimine-functionalized silver nanoparticle interaction/binding with *C. albicans* cell surface biomolecules. Although, a quantitative approach is needed to better understand the interaction dynamics in order to formulate selective surface tuned nanoparticle for selective antifungal activity.

## Introduction

Nanoparticles (NPs) are a focus of materials research since they often exhibit different properties from those of their bulk material counterparts (Baun et al., 2008). Ecological and health considerations have been raised in relation to the interaction of NPs with the environment (Gupta and Gupta, 2005; Brunner et al., 2006). For example, the NPs may build up on cell surfaces (Zhu et al., 2009; Schwegmann et al., 2010), cause the loss of cellular mobility (Baun et al., 2008), produce membrane translocation (Wong-Ekkabut et al., 2008), and facilitate DNA damage (Zhang et al., 2010). Many of these biological effects have been noted with microorganisms (Li et al., 2010) in wastewater treatment plants (Kiser et al., 2010), the natural environment, and the human digestive system (Zhang et al., 2009).

Silver nanoparticles (Ag-NPs) have been considered for use in sensors, cosmetics, paint/varnishes, and biomedical products due to their unusual optical, electronic, and catalytic properties (Al-Rajhi et al., 2022; Salem and Fouda, 2021; Salem et al., 2022a,b; Elakraa et al., 2022). Their unique physicochemical properties have made them one of the most commercialized nanomaterials (Chen and Schluesener, 2008). Polyethyleneimine is a polymer with polycationic and hydrophilic properties that is used for DNA transfection of mammalian cells, as a membrane perforating agent, and as a coating material in the biomedical and industrial fields. In our previous studies, we have documented the ultrafast, size-controlled, and water-dispersible synthesis of polyethyleneimine-functionalized silver and gold nanoparticles based on the polyethyleneimine molecular weight (Pandey et al., 2017; Tiwari et al., 2020). Other than polyethyleneimine, silica-containing organic polymers such as 3-aminopropyltrimethoxysilane and 3-glycidoxytrimethoxysilane and organic reducing agents such as formaldehyde and cyclohexanone are used to synthesize mono-, bi-, and trimetallic noble metal nanoparticles (Pandey et al., 2020). Thus, imine groups in PEI facilitate the nucleation and act as stabilizers (Tiwari et al., 2020).

There are few studies involving the adsorption of NPs on cell surfaces; quantitative studies involving particle adsorption kinetics have also not been previously considered. Wilhelm et al. considered NP binding on cell surfaces *via* a pseudo first-order kinetic model (Wilhelm et al., 2002). Other research teams used this kinetic model to understand NP interactions with human cells (Cho et al., 2009; Zhang et al., 2010). It should be noted that a pseudo-first-order model does not indicate the mechanism of action. Efforts are needed to develop mechanistic models for NP adsorption to microbial cells. The surface charge and size of Ag-NPs as well as the type of bacterial species determine the potency of the antimicrobial effect; for example, smaller-diameter Ag-NPs often demonstrated more potent antibacterial activity. Since the surfaces of bacteria are negatively charged, positively charged Ag-NPs exhibited greater antimicrobial activity than negatively charged Ag-NPs. Despite significant research activity involving Ag-NPs, the relationship between antimicrobial activity and Ag-NP/bacterial surface interactions is not fully elucidated.

Cells contain various biomolecules (e.g., NADH, flavins, and proteins) that fluoresce under UV irradiation and show intrinsic autofluorescence at a specific excitation wavelength. The three aromatic amino acids tyrosine, tryptophan, and phenylalanine exhibit excitation maxima of 280, 275, and 260 nm, respectively; the emission maxima are 350, 300, and 280 nm, respectively. In addition, several proteins become expressed on the cell surface, act as receptors for exogenous ligands, and play a potential role in pathogenesis. Since these proteins are excited and auto-fluoresced on UV irradiation at 280 nm, they can be used as a fluorescent molecular probe to monitor the interaction between nanoparticles and cells. It is well established that physical characteristics such as shape, surface area, particle size, and surface charge are parameters that are associated with interactions between nanoparticles and microorganisms (Karakoti et al., 2006). Fluorescence spectroscopy has been extensively utilized for molecular structure and function studies in chemistry and biochemistry. However, its use for monitoring the molecular-level interaction phenomena between nanoparticles and living cells at the nano-bio interface has not been considered as extensively. Herein, we exploited fluorescence spectrophotometry to understand the interaction dynamics of polyethyleneimine-functionalized silver nanoparticles at the *C. albicans* cell bio-interface. Furthermore, the agar well diffusion, micro broth dilution, flow cytometry, and compound light microscopy were used for the elucidation of the antifungal mechanism of the synthesized silver nanoparticles.

## Materials and methods

### Fungal strain, culture media, and growth conditions

*Candida albicans* (ATCC 90028, American Type Culture Collection, Manassas, VA, United States) was received from the Department of Microbiology, Institute of Medical Sciences, Banaras Hindu University, Varanasi, India. After collection, the culture was revived in YPD (Yeast extract peptone) broth (supplemented with 1% glucose) at neutral pH (7.2) and preserved in 25% glycerol at-80oC for further investigation. Polyethyleneimine, silver nitrate (AgNO_3_), calcofluor white, bovine serum albumin (BSA), tryptophan, tyrosine, amphotericin B, and other routine chemicals were obtained from Sigma Aldrich Chemicals Private Limited (Bangalore, Karnataka, India). RPMI (Rosewell Park Memorial Institute media) and other media constituents were obtained from Hi-Media Laboratories Ltd. (Mumbai, Maharashtra, India). Plasticware was obtained from Tarsons Products Limited (Kolkata, West Bengal, India). The solvents, including ultra-purified water, were obtained from Merck Life Science Private Limited (Bangalore, Karnataka, India). All of the reagents were of analytical grade. The generated experimental data were plotted and analyzed using Origin 8.5 software (Origin Lab Corporation, Northampton, MA, United States).

### Synthesis and physical characterization of PEI-functionalized silver nanoparticles

The polyethyleneimine (PEI)-functionalized Ag-NPs were synthesized as reported previously with slight modifications (Tiwari et al., 2020). In a 2 mL glass vial, ethylene glycol diacetate (8–10%) and a methanolic solution of 1-vinyl 2-pyrrolidone (VPP, 50 μL from a 50 mM stock solution) were combined; a methanolic solution of AgNO_3_ (10 mM solution), cyclohexanone (20 μL), and PEI (16.4 mg/mL; 40 μL) was added to the mixture. The mixture was thoroughly agitated on a vortex mixer for 30 s and subsequently placed in a microwave oven; for complete reduction of the Ag + cations, the 5 s cycle was repeated four to six times. The resulting mixture exhibited a deep brown color, which denoted the creation of PEI-f-Ag-NP. The nanoparticles were reconstituted in ultra-pure water to conduct additional characterization studies.

The synthesized Ag-NPs were characterized using a U-2900 UV–Vis spectrometer (Hitachi, Tokyo, Japan); 200–800 nm wavelengths were used for data collection. Transmission electron microscopy of the Ag-NPs was performed using a Tecnai G2 20 Twin (FEI, Hillsboro, OR, United States); the sample was diluted in methanol and then drop-cast over 300 mesh carbon-coated copper grids. Zeta potential analysis of the samples was accomplished with a Zetasizer instrument (Malvern Panalytical, Malvern, UK). X-ray diffraction analysis of synthesized nanoparticles was performed by forming a film of the sample on a glass coverslip and drying the sample at 70°C for 2 h. Spectra were recorded using a MiniFlex 600 X-ray diffractometer (Rigaku, Tokyo, Japan).

### Assessment of antifungal activity and MIC determination of PEI-functionalized silver nanoparticles

The agar well diffusion method was utilized to evaluate the antifungal activity of functionalized silver nanoparticles. In short, log-phase cells of *C. albicans* in RPMI media were adjusted to 10^6^ cfu/mL, swabbed on MHA (Muller Hinton ager) plates with a sterile cotton swab, followed by surface drying for 10 min. Agar wells were formed through the wide mouth of the sterile pipette tip. A 20 μL of constituted silver nanoparticles was poured into an agar well and incubated at 28°C for 48 h.

The broth microdilution method was used to obtain the MIC (Minimum Inhibitory Concentration) value of PEI functionalized Ag-NPs against chosen fungus; as described previously, a sterile flat-bottom 96-well microtiter plate was used for the study (Andrews, 2001; Cavaleri et al., 2005; Li et al., 2005). In short, log-phase cells of *C. albicans* grown in RPMI broth were centrifuged, resuspended in a fresh RPMI medium, and adjusted 0.5 MacFarland standard fungal suspension for MIC determination measurements (McFarland, 1907). An active suspension of 50 μg/mL of functionalized Ag-NPs was prepared in ultra-pure water and diluted serially; the final concentration was obtained as 0.43–50 μg/mL. Hundred microliter of the fungal suspension was then dispensed into each well. Amphotericin B was used as a positive control in this study. The microtiter plate was incubated at 28°C for 24 h; a visual demonstration of complete fungal inhibition (i.e., a visually clear well) was assigned the MIC value. Subsequently, a 5 μL suspension from a visually clear well was sub-cultured on the YPD plates for 24 h and fungal growth was noted. To ensure the accuracy and minimizing the handling errors, all measurements were performed in triplicate.

### Fluorescence spectroscopy

The log phase culture of *C. albicans* in RPMI media was obtained; it was centrifuged at 3500 rpm for 4 min, washed twice with PBS (Phosphate Buffer Saline) to remove media traces, and resuspended in phosphate buffer. The least cell count (5×10^2^ cells/mL) was adjusted in a 1 cm quartz cuvette to avoid an inner filter effect. A F-7000 fluorescence spectrophotometer (Hitachi, Tokyo, Japan) was used to obtain fluorescence emission (FL emission) spectra at room temperature. The routine excitation wavelength for proteins (λ = 280 nm) was used to obtain the emission spectra of *C. albicans* cells and bovine serum albumin V (BSA) (10 μL of 1 μg/mL). In comparison, λ = 270 nm was utilized for tryptophan and tyrosine (10 μL of 1 μg/mL) in phosphate buffer (pH 7.2). The slit width values for excitation and emission were set to 5.0/10 nm, respectively. The 3D FL spectra were recorded at λex/em = 280/300 nm matrix. The number of scans was 20 at 10 nm per scan increment. Further, FL spectra were recorded as a control to observe the effect of UV exposure on the *C. albicans* cells. The measurements were made in triplicate in phosphate buffer (pH 7.2).

### Intracellular ROS generation assay

To investigate the antifungal mechanism, the intracellular ROS level in treated cells was evaluated using an oxidation-sensitive fluorescent dye called 2′,7′-dichlorodihydrofluorescein diacetate (H_2_DCFDA, Molecular Probes, Eugene, OR, United States). The cells were washed with PBS buffer and incubated with PEI-f-Ag-NPs for 2 h at 28°C. After centrifugation, the cells were incubated with H_2_DCFDA for 30 min in the dark and washed twice. Ten thousand counts were collected; the FL intensity was measured using a cytoFLEX LX flow cytometry instrument (Beckman Coulter Inc., Pasadena, CA, United States) (Seong and Lee, 2018).

## Results and discussion

The antifungal action of silver nanoparticles is a two-step process; the first step involves the nonspecific binding (or adsorption) of cationic nanoparticles on the cell surface. The second step involves the execution of antimicrobial action. Herein, we reported the binding dynamics of PEI-functionalized nanoparticles on the *C. albicans* cell surface *via* FL quenching phenomena by using cell surface proteins as fluorescent molecular probe. The second part of the study involves the elucidation of antifungal mechanism of functionalized silver nanoparticles.

### Physical characterization of synthesized polyethyleneimine functionalized silver nanoparticles

**Figure 1:**
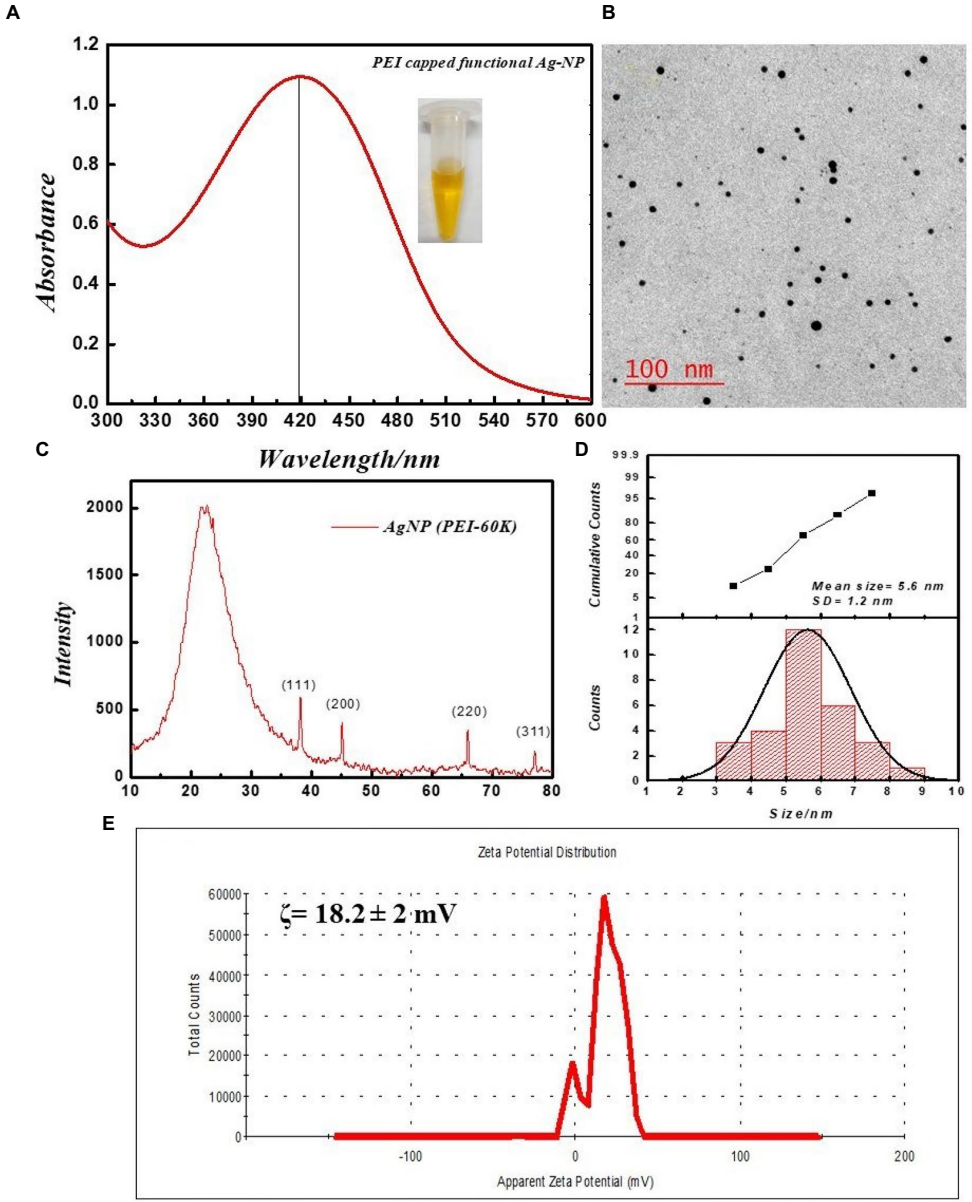
Physical characterization of PEI-f-Ag-NPs. **(A)** UV–VIS spectra: inset dispersed in water, **(B)** respective TEM image, **(C)** XRD diffractogram, **(D)** size distribution histogram, and **(E)** Zeta potential distribution.

The silver nanoparticle was stabilized and functionalized by branched polyethyleneimine with a molecular weight of 60,000 Da. The synthesized silver nanoparticles were characterized using UV–Vis spectroscopy, TEM, zeta potential, and X-ray diffraction methods. Figure 1A shows the UV–Vis absorption spectra at 419 nm; these results are consistent with the characteristic absorption band of Ag-NPs from surface plasmon resonance (SPR). The size of the PEI-functionalized silver nanoparticles in a water solution was recorded using TEM (Figures 1B,D). The average size of the PEI-f-Ag-NPs was 5.6 ± 1.2 nm (mean ± SD) with a spherical shape; the corresponding histograms revealed that the particle size distributions were between 3–9 nm and revealed near monodispersed distribution (Figure 1D). In addition, the ζ potential of silver nanoparticles was recorded as 18.2 ± 2 mV, indicating high stability (Figure 1E). The colloidal stability of synthesized silver nanoparticles was ensured by interparticle electrostatic repulsion. The XRD pattern was recorded for the 2θ angle from 5 to 80 degrees. The generated diffractogram was compared with the standard powder diffraction card of JCPDS, silver file No. 04–0783. The observed Peaks at 2θ degrees of 38.116, 44.583, 64.418, and 77.438 degrees in the diffractogram were noted to correspond to (hkl) values of the (111), (200), (220), and (311) planes of silver as indicated in Figure 1C. Thus, the XRD study confirmed that the functionalized silver nanoparticles possess a face-centered cubic crystal structure.

### Antifungal activity and minimum inhibitory concentration of PEI-f-Ag-NPs

**Figure 2:**
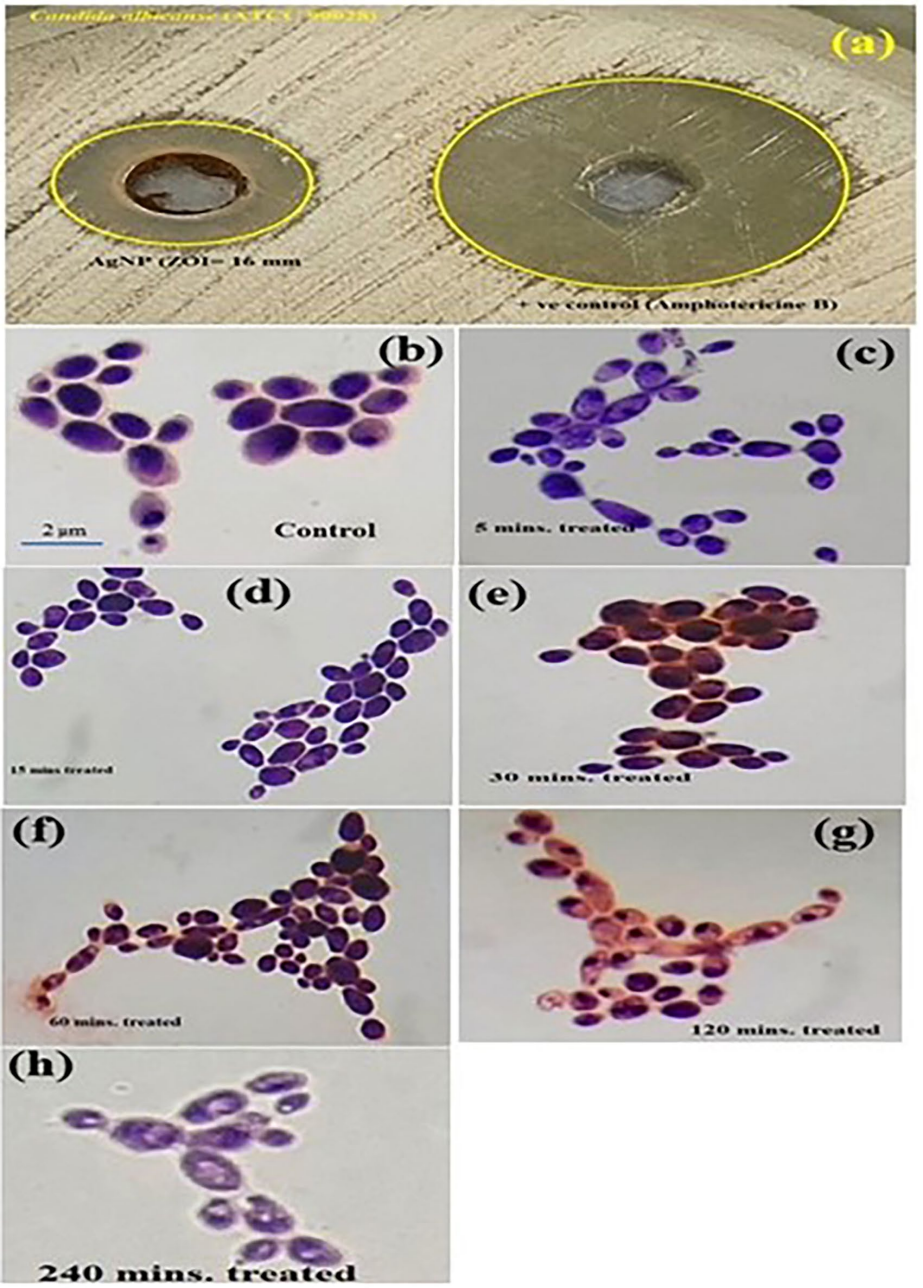
**(A)** Image shows the zone of inhibition of *C. albicans* by PEI-f-Ag-NPs. Compound light microscopy photographs of PEI-f-Ag-NPs treated *C. albicans* cells for various times: **(B)** untreated control; **(C)** treated for 5 min; **(D)** 15 min; **(E)** 30 min; **(F)** 60 min; **(G)** 120 min; and **(H)** 240 min.

The synthesized PEI-f-Ag-NPs were evaluated for their antifungal activity by the agar well diffusion method. The 16 ± 2 mm zone of inhibition indicated potent antifungal activity against *C. albicans* (Figure 2A). Further, the observed MIC value of PEI-f-Ag-NPs against *C. albicans* was 5 ± 0.5 μg/mL. Furthermore, the cells were treated with PEI-f-Ag-NPs at their MIC value (5 μg/mL) in a time-dependent manner (5, 15, 30, 60, 120, and 240 min); the cell morphology was observed using a compound light microscope (Figures 2B–H). The cell wall/membrane was damaged (a visual pit hole formation) over time and showed a strong antifungal activity within 30 min of treatment (a reduction of ~70% cell viability). Complete damage to cellular architecture was observed at 240 min of treatment.

### Fluorescence spectroscopy investigation of cell-nanoparticle binding

**Figure 3:**
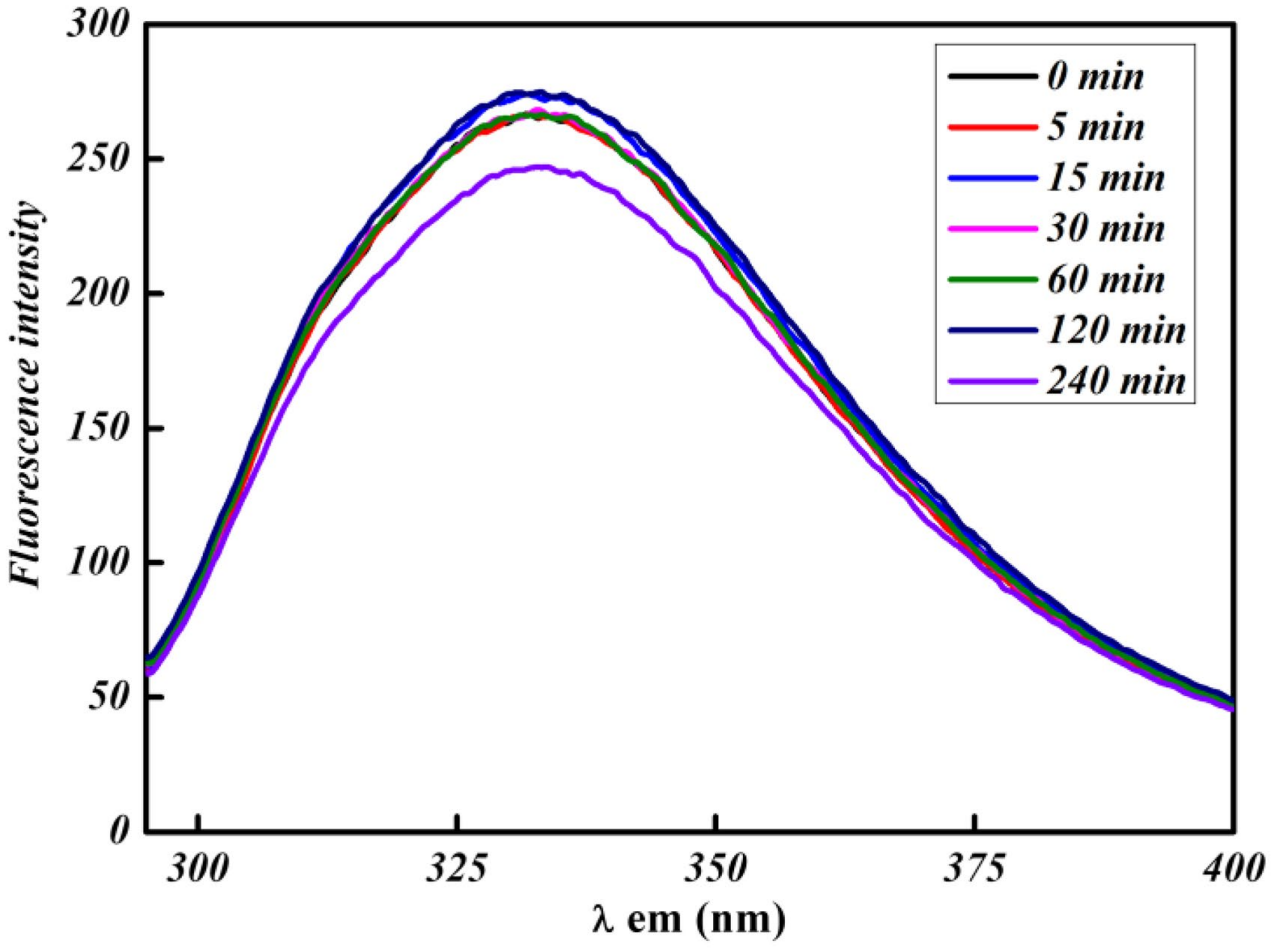
UV exposure effect at various times on bare *C. albicans* cells intrinsic protein fluorescence.

**Figure 4:**
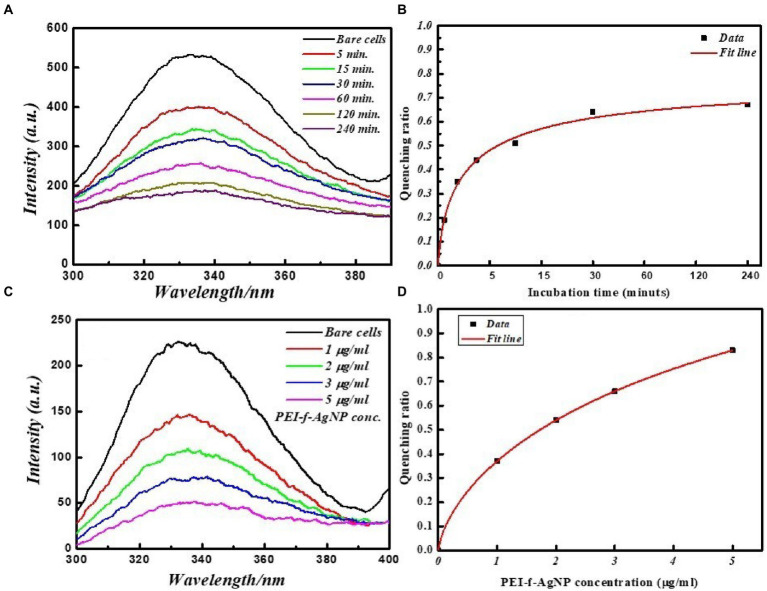
FL emission spectra of *C. albicans* cell surface proteins at various incubation times with and without PEI-f-Ag-NPs. **(A)** 2D fluorescence emission spectrum, **(B)** FL quenching ratio by PEI-f-Ag-NPs at various incubation times, **(C)** FL emission spectra of *C. albicans* cell surface proteins with increasing concentration of PEI-f-Ag-NPs, and **(D)** FL quenching ratio with an increasing PEI-f-Ag-NPs concentration.

All of the FL spectroscopic experiments were performed at room temperature and in phosphate buffer having neutral pH. The number of *C. albicans* cells and the concentration of functionalized silver nanoparticles per ml were kept low to avoid any inner filter effect. A control experiment was performed by exposing cells for a short time to evaluate the effect of UV light on proteins, which shows a slight effect (Figure 3). The FL result showed that PEI-f-Ag-NPs quenched the autofluorescence of *C. albicans* surface protein with an increasing time of incubation (incubated for 5, 15, 30, 60, 120, and 240 min) (Figure 4A); a similar result was noted with an increasing concentration (1, 2, 3 and 5 μg/mL) (Figure 4C). The characteristic FL quenching by PEI-f-Ag-NPs indicates strong binding with *C. albicans* cells at the nano-bio interface. The autofluorescence of the *C. albicans* cell surface protein quenching ratio by PEI-f-Ag-NPs was assessed with an increasing incubation time and concentration of silver nanoparticles, which showed rapid binding (Figures 4B,D). The plot of the quenching ratio against incubation time indicated multiple binding sites on the *C.albicans* surface for PEI-f-Ag-NPs and binding saturation over time. FL quenching achieves a steady state at 60 min of incubation because of binding site saturation (Figure 4B). Similarly, with an increasing concentration of PEI-f-Ag-NPs, the FL quenching ratio was reached at a steady state at 5 μg/mL. This result can be interpreted as saturation of binding sites on the cell surface (Figure 4D). A similar saturation effect was previously described by Wilhelm et al. (2002); however, they studied HeLa cells to understand the interaction dynamics of anionic super magnetic iron oxide nanoparticles. Further, Zheng et al. reported that smaller hematite nanoparticles had faster adsorption than larger ones on the *E. coli* surface (Zhang et al., 2010). These studies strongly support the rationality and underlying binding mechanism and the adsorption dynamics of functionalized silver nanoparticles on *C. albicans* cells surface.

### Mechanism of FL quenching

Fluorescence involves short-term emission of light (of no greater than 10^−8^ s after excitation), which is associated with the absorption of electromagnetic radiation (e.g., infrared visible, or ultraviolet light) (Monici, 2005). The term “autofluorescence” is used to differentiate the intrinsic fluorescence associated with cells and tissues from the fluorescence associated with exposing samples to exogenous fluorescent markers that attach to cell and tissue components. The autofluorescence properties of biomolecules have been previously utilized to identify pathogenic microorganisms and cellular biomolecules (Richards-Kortum and Sevick-Muraca, 1996; Fusi et al., 2002).

FL quenching describes phenomena that are associated with a reduction in the FL intensity (Monici, 2005). These phenomena can be associated with intermolecular interactions (e.g., energy transfer, molecular rearrangements, and excited-state reactions); moreover, the quenching phenomena can exhibit dynamic (e.g., related to diffusive encounters between the quencher and the fluorophore within the excited state lifetime) or static (e.g., related to complex formation between the quencher and the fluorophore in the ground state (Monici, 2005). *C. albicans* exhibits an outer rigid cell wall structure covered by the cell membrane, which is composed of complex sugar moieties such as β-glucans and several anchored proteins that act as receptors for exogenous ligands. When excited with UV irradiation, these surface-anchored proteins fluoresce due to the aromatic amino acid tyrosine, tryptophan, and phenylalanine and act as a binding site for PEI-f-Ag-NPs. The fluorescence quenching data were correlated with the time-dependent kill kinetics of the cell; the results indicate that cellular architecture was destabilized as treatment time increased from 5–240 min (Figures 2, 4).

**Figure 5:**
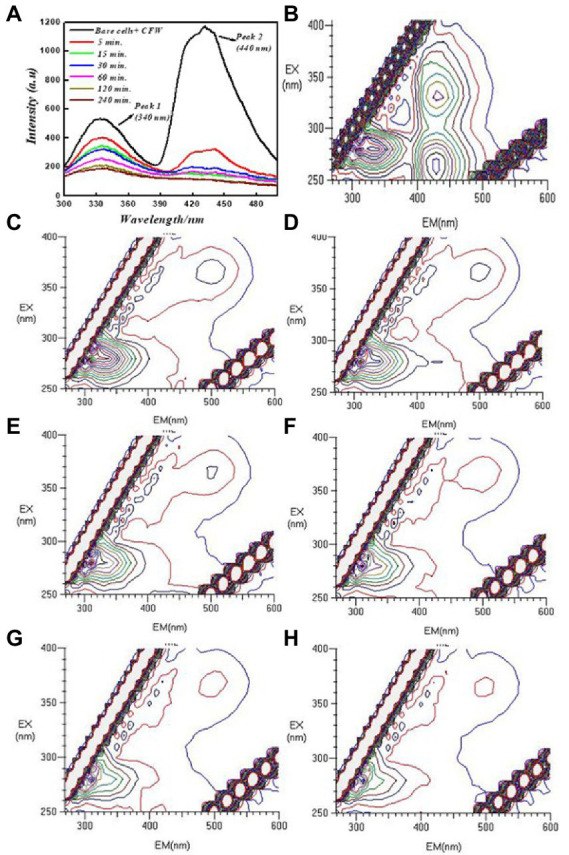
2D and 3D FL emission spectra of calcofluor white-stained *C. albicans* cells at various incubation times treated with PEI-f-Ag-NPs and without treatment: **(A)** 2D FL emission spectrum, **(B)** 3D FL spectrum without PEI-f-Ag-NPs (cell control), **(C)** 3D FL spectrum involving incubation with PEI-f-Ag-NPs for 5 min, **(D)** 3D FL spectrum involving incubation with PEI-f-Ag-NPs for 15 min, **(E)** 3D FL spectrum involving incubation with PEI-f-Ag-NPs for 30 min, **(F)** 3D FL spectrum involving incubation with PEI-f-Ag-NPs for 60 min, **(G)** 3D FL spectrum involving incubation with PEI-f-Ag-NPs for 120 min, and **(H)** 3D FL spectrum involving incubation with PEI-f-Ag-NPs for 240 min.

To better understand the interaction of PEI-f-Ag-NPs with molecules other than proteins, the cells were stained with Evans blue and calcofluor white for 15 min and then washed with water; 2D and 3D fluorescence spectra were obtained in the presence of PEI-f-Ag-NPs at various times. Calcofluor white (CFW) is a fluorescent blue-colored dye that is used for diagnosing fungal onychomycosis, which binds to the 1–3 and 1–4, β-linkage of chitin and cellulose in fungal, plant, and algal cell walls. The excitation wavelength for CFW is 380 nm; the emission maxima are between 440 and 475 nm (Shetty et al., 2019). The results showed that PEI-f-Ag-NPs quenched the fluorescence of CFW within 5 min; however, complete quenching was recorded at 120 min of incubation (Figure 5).

The function of electrostatic interactions between the cell surface and charged nanoparticles has previously been described for cellular uptake (Farquhar, 1978; Ghinea and Simionescu, 1985; Mutsaers and Papadimitriou, 1988). Cationic liposomes were noted to bind more efficiently than anionic and neutral ones (Lee et al., 1993; Miller et al., 1998; Chenevier et al., 2000). Cationic ferritin particles were noted to uniformly adsorb to the plasma membrane of fixed mammalian cells; this phenomenon was attributed to the many large anionic domains on the cell surface (Farquhar, 1978; Mutsaers and Papadimitriou, 1988). These previous findings, strongly supporting our approach to study the inter-molecular interactions at nano bio interface. Since, our present study was dedicated to qualitative investigation of functionalized silver nanoparticle binding on living system surface biomolecules.

**Figure 6:**
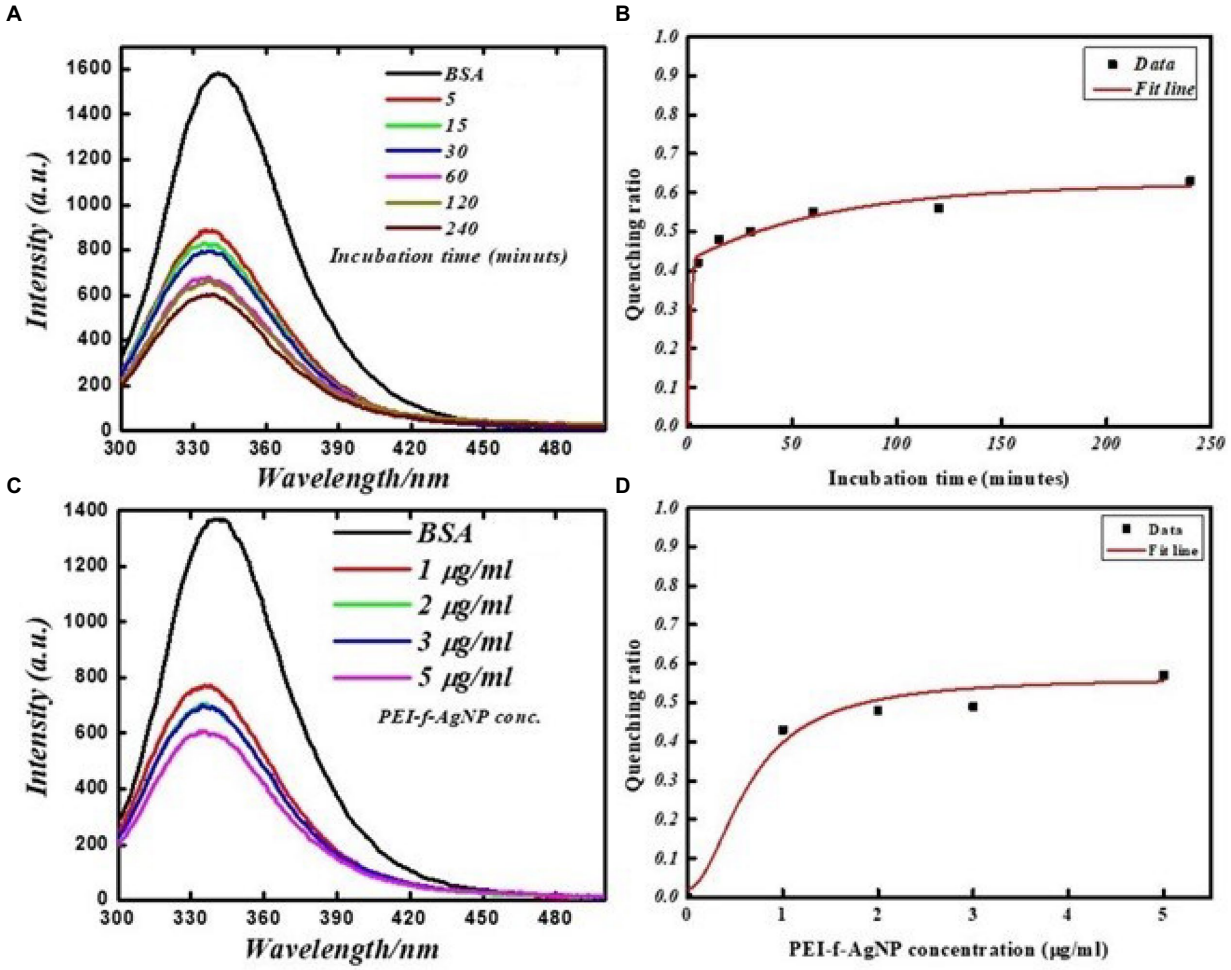
FL emission spectra of BSA protein at various times with and without PEI-f-Ag-NPs: **(A)** fluorescence emission spectrum at various incubation times, **(B)** FL quenching ratio at various incubation times, **(C)** FL emission spectra with increasing concentration of PEI-f-Ag-NPs, and **(D)** FL quenching ratio with increasing PEI-f-Ag-NPs concentration.

Additional studies were carried out to investigate the specific binding of PEI-f-Ag-NPs with the protein BSA and selected aromatic amino acids, tryptophan and tyrosine residues. The FL emission of the BSA-PEI-f-Ag-NP system was measured in phosphate buffer at neutral pH and room temperature. The BSA was incubated with functionalized silver nanoparticles at various times and concentrations similar to *C. albicans* cells. BSA (Bovine serum albumin) is comprised of 607 amino acids (66 kDa), with 24 tyrosine residues and two tryptophan residues (Ray et al., 2009). Tyrosine and tryptophan residues exhibit intrinsic fluorescence; the tryptophan emission dominates the UV fluorescence spectra for BSA (Ray et al., 2009). The results indicated that the silver nanoparticles bound and quenched FL of BSA with increasing contact time and PEI-f-Ag-NP concentration similar to *C. albicans* cells with a comparable dynamic (Figure 6). The characteristic emission band at 340 nm decreased as increment in concentration of PEI-f-Ag-NPs; this correlation suggested a strong interaction between nanoparticles and BSA. Mariam et al. performed a similar study in which 10 mg/mL BSA and 90–812 mL Ag-NPs were used (Mariam et al., 2011). In another study, Huang et al. described the interaction between gold nanoparticles and BSA (Huang et al., 2014). The quenching ratio was studied; with an increase in incubation time and concentration, the binding of BSA on nanoparticles achieved a steady state (Figure 6); this result indicated the saturation of vacant binding sites on silver nanoparticles. Additional studies are underway to estimate the number of bindings of BSA molecules on the surface of a single PEI functionalized silver nanoparticle.

**Figure 7:**
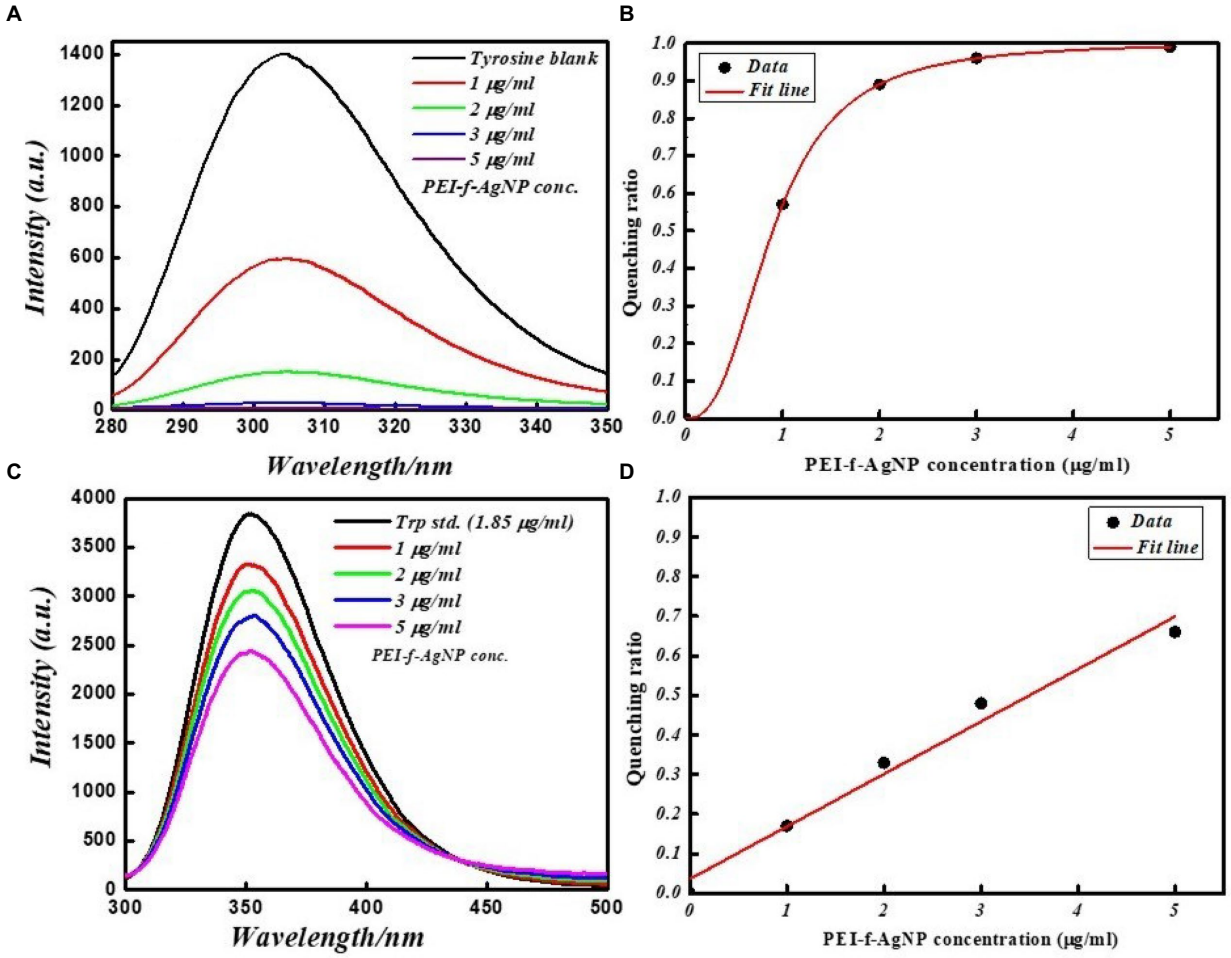
FL emission spectra of tyrosine and tryptophan amino acid standards at various concentrations with and without PEI-f-Ag-NPs: **(A)** fluorescence emission spectrum of tyrosine, **(B)** FL quenching ratio by PEI-f-Ag-NPs of tyrosine at various concentrations, **(C)** FL emission spectra of tryptophan at various concentrations of PEI-f-Ag-NPs, and **(D)** FL quenching ratio at various PEI-f-Ag-NPs concentration.

The interaction of PEI-f-Ag-NPs with tryptophan and tyrosine residues was investigated in a more detailed study as a standard fluorophore. Tryptophan and tyrosine exhibit intrinsic emission upon excitation on 280 and 270 nm, respectively. Figure 7 shows the FL emission spectra and quenching ratio of tryptophan and tyrosine recorded with various PEI-f-Ag-NPs concentrations. The fluorescence intensity of tryptophan and tyrosine was noted to decrease as the silver nanoparticle concentration was increased. Silver nanoparticles were able to interact with tryptophan and tyrosine, thus quenching the fluorescence intensity. PEI-f-Ag-NPs exhibit strong interactions with tyrosine compared to tryptophan residues, indicating a strong probability for interactions with tyrosine-rich proteins. The presented work conclusively, demonstrating a complex formation between cell surface anchored proteins and functionalized silver nanoparticle and quenched the fluorescence of protein with a saturation kinetic model. At this point, it is obvious to study, whether, functionalized nanoparticles are binding on specialized cell surface domains or binding uniformly. Depending on the type of cells, the distribution and nature of surface biomolecules varies. Thus it is interesting to note the cell and functionalizing agent specific binding kinetics of nanoparticle interaction with cells.

### Measurement of binding constant and number of binding sites

The modified Stern-volmer equation (double logarithm regression curve) can be used to obtain the binding constant as well as the number of binding sites/macromolecules, in which it is presumed that PEI-f-Ag-NPs bind independently to cell surface macromolecules (equivalent sites) (Hu et al., 2004; Xu et al., 2011).

$$\log\;\left[\left(F_{0}-F\right)/F\right]=\log\;Kb+n\;\log\;\left[Q\right]\text{.}$$

**Figure 8:**
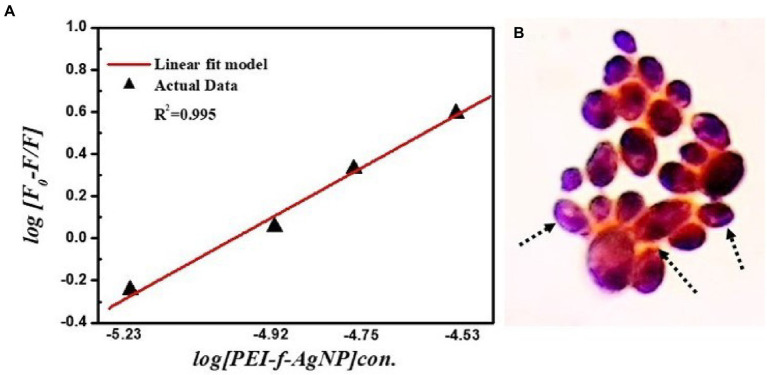
**(A)** Binding constant (Kb) and number of binding sites (n) for PEI-f-Ag-NP with *C. albicans*; **(B)** Schematic depicts adsorbed PEI-f-Ag-NPs on the surface of *C. albicans* cells.

In this equation, Kb represents the binding constant, F_0_ represents fluorescence intensity without a quencher, F represents fluorescence intensity with a quencher, and n represents the number of binding sites (Xu et al., 2011). The number of binding sites and the binding constant were obtained for *C. albicans* cells. Plots of log [(F_0_–F)/F] versus log [PEI-f-Ag-NP] are shown in Figure 8A. The values of Kb and n were determined from the intercept on the *y*-axis and the slope of this plot, respectively. The Kb and *n* values of PEI-f-Ag-NPs for *C. albicans* cells were 1× 10^5^ M^−1^ and 1.01, respectively (Figure 8A). The high binding constant values shows strong binding between PEI-f-Ag-NPs and *C. albicans cell* surface. Also, it is indicated that each interacting cell anchored protein can bind to 1.0 PEI-f-Ag-NP at a time. However, this phenomenon may be different for various types of cells because different types of cells have variable biomolecules composition with distinct physico-chemical properties. The apparent binding constant is dependent on several physical parameters, including the concentration of Ag-NPs and temperature; further, it is also dependent on the size-shape and surface tuning parameters of the Ag-NPs, which in turn are dependent on the synthesis protocol (Dasgupta et al., 2016). It should be noted that the apparent association constant is also dependent on the temperature; the role of temperature was not considered in this study as the focus of the study was to determine the nature and dynamics of the interaction. The binding constants (Kb) indicate that the affinity of PEI-f-Ag-NPs for BSA is very low in comparison to the reported binding constants, which range from 10^4^ to 10^8^; this finding supporting previous reports that serum albumin contains few binding sites for endogenous and exogenous ligands, which are commonly bound in a reversible manner (Rahman and Sharker, 2009). Hence, the above result concluded that, PEI-f-Ag-NPs have a strong binding tendency with complex cell wall polysaccharides and surface anchored proteins. The microscopic demonstration of surface binding of PEI-f-Ag-NPs on the *C. albicans* cell can be seen in Figure 8B.

### Antifungal mechanism of PEI functionalized silver nanoparticles

**Figure 9:**
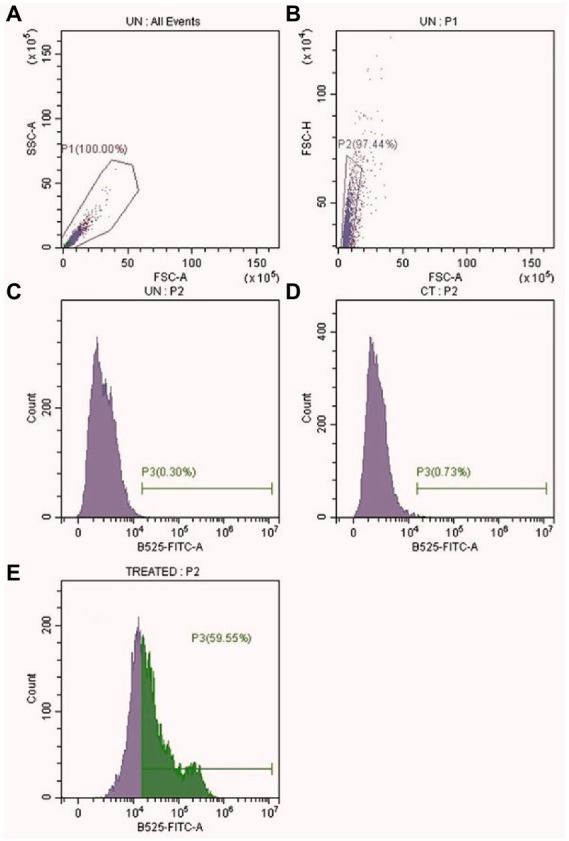
Intracellular ROS level in PEI-f-Ag-NPs treated *C. albicans* cells. **(A)** All events **(B)** Selected events (10000) **(C)** Unstained Control **(D)** Stained Control **(E)** PEI-f-Ag-NPs treated.

**Figure 10:**
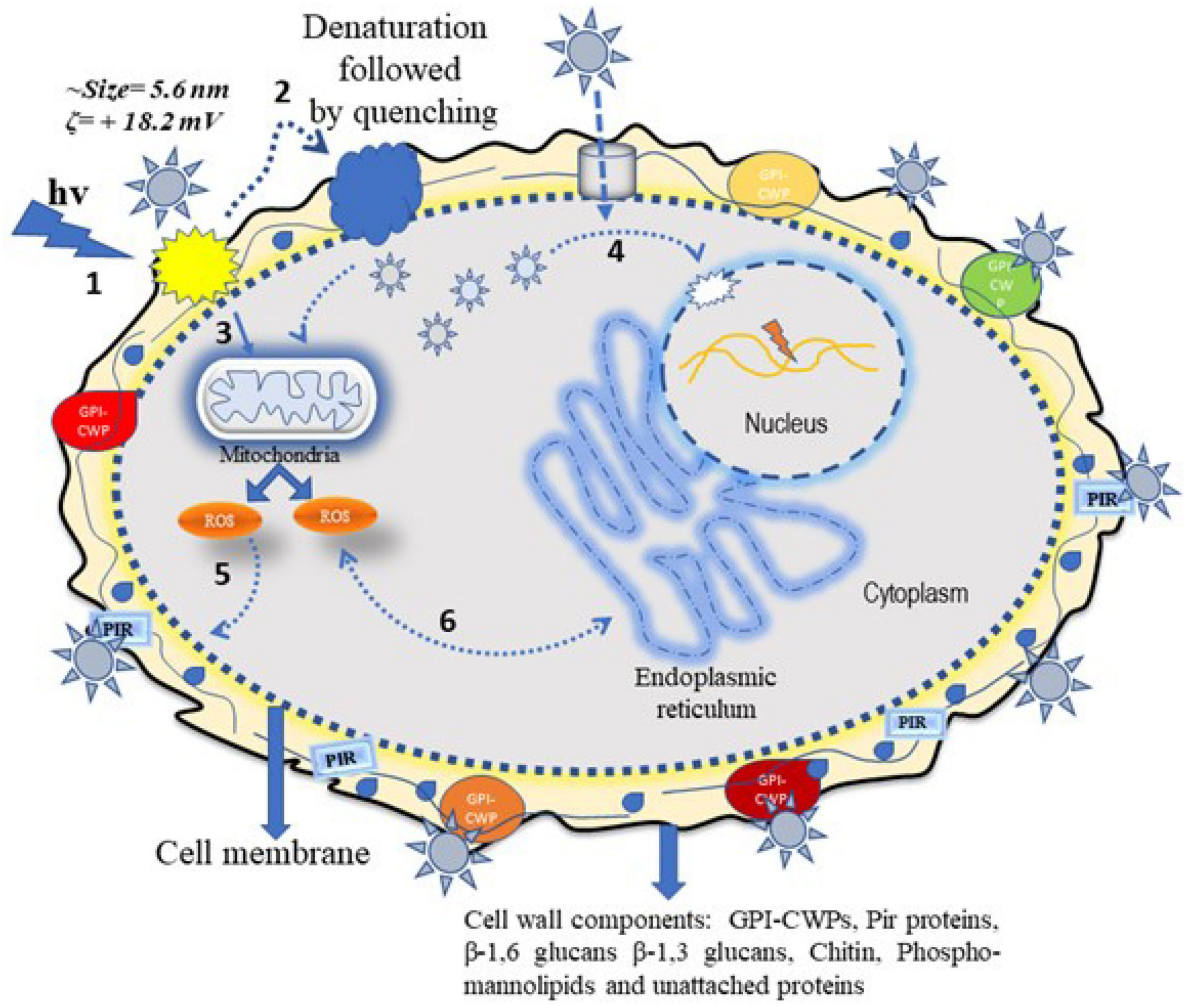
Schematic showing the possible antifungal mechanism of polyethyleneimine-functionalized silver nanoparticles. The silver nanoparticle interacts with *C. albicans* cells *via* two different pathways, which can be direct or indirect. In the direct pathway, the cationic Ag-NPs interact electrostatically with surface structural proteins and phosphor-mannolipids; these processes induce electrostatic stress followed by ROS generation and inactivation of cytoplasmic proteins. In addition, nanoparticle-generated stress induces mitochondrial ROS inside the cell, which damages cytoplasmic biomolecules such as proteins and DNA through oxidation. The ROS can also damage the cell membrane through lipid peroxidation, resulting in membrane perforation and eventual cell death (steps 1, 2, 3, 5, and 6) as indicated in the diagram. The indirect action involves silver nanoparticles passing through the cell wall/membrane *via* water channels or other channels and becoming internalized inside the cell. These silver nanoparticles disintegrate the cytoplasmic biomolecules (e.g., structural proteins and enzymes) and nuclear DNA, collapsing cell cytoarchitecture and resulting in cell death (step 4) as indicated in the diagram.

Evidence of the antimicrobial use of silver ions appears in the historical record (Alexander, 2009). The antimicrobial mechanism of silver, either in a free ionic form or in a nanoparticle form, is not clearly defined. The known mechanism of silver indicates a multidimensional action to disrupt many fundamental functions of the microbial cell (Rai et al., 2012; Mikhailova, 2020). The antimicrobial activity of Ag-NPs is dependent on their shape and size; some of the observed effects can be associated with the release of Ag^+^ ions from the nanoparticle surface (Kędziora et al., 2018; Tang and Zheng, 2018). The cationic Ag-NPs exhibit a high affinity for the anionic surface of microorganisms (Mikhailova, 2020). Once nanoparticles adhere to the microbial surface, they initiate a “pitting” effect in the cell wall, which is associated with architectural collapse; this process results in leakage of intracellular contents, dissipation of the transmembrane potential, as well as denaturation to membrane-associated proteins (Tang and Zheng, 2018; McNeilly et al., 2021). Silver nanoparticles also initiate the production of toxic intracellular ROS that result in cell damage (McNeilly et al., 2021). The antifungal mechanism in PEI-f-Ag-NPs treated *C.albicans* cells in this work is associated with a huge generation of endogenous ROS accumulation and pit hole formation in cell wall (Figure 9). As seen in Figure 8B, it appears that intracellular ROS was the main cause of cell damage. Our previous study found that PEI-f-AgNP-1 interacts with *Rhizopus arrhizus* sporangiospores by generating stress, followed by the generation of ROS species, which ultimately damages the spore wall (Tiwari et al., 2022). A schematic representation of antifungal mechanism associated with the PEI-f-Ag-NPs against *C. albicans* cells can be seen in Figure 10.

## Conclusion

The study sought to understand how the physicochemical properties of silver nanoparticles affect their antimicrobial activity. When the nanoparticles are added to the microbe-containing medium, they first encounter the surface of the microbe. To understand the nanoparticle interactions with the *C.albicans* cell surface, we used polyethyleneimine-functionalized silver nanoparticles with a size of 5.6 nm having a + 18 mV zeta potential. The PEI-functionalized silver nanoparticles have shown potent biocidal activity with a MIC value of 5 μg/mL. The study showed that PEI-f-Ag-NPs interacted strongly with proteins and cell wall polysaccharide components within 60 min of treatment. The binding constant (Kb = 1.0 × 10^5^ M^−1^) as well as the number of binding sites (*n* = 1.01) were high for the cell surface proteins. Furthermore, the intrinsic fluorescence was quenched by PEI-f-Ag-NPs, indicating a strong interaction with tyrosine residues rather than tryptophan residues. This study provides an approach to qualitatively understand the molecular-level interactions of metal nanoparticles and biomolecules by exploiting cell proteins as a molecular probe. Understanding the interaction of NPs and surface proteins will facilitate the development of smart antimicrobial nanomaterials with enhanced biocidal properties against many types of microorganisms.

## Future directions

The presented work investigated only qualitative static interactions *via* fluorescence spectroscopy of silver nanoparticles with *C. albicans* surface proteins. A qualitative and quantitative investigation involving binding kinetics, binding isotherm studies, and cell type-dependent interaction studies is underway; these studies are needed to understand molecular-level interactions of functionalized metal nanoparticles at the nano-bio interface to avoid cytotoxicity concerns, formulation of nano antimicrobials, and harness the antimicrobial potential of metal nanoparticles.

## Data availability statement

The raw data supporting the conclusions of this article will be made available by the authors, without undue reservation.

## Author contributions

AT conceived and designed the experiments. AT and MG conducted the sample preparation, conducted the experiment, and analyses. AT and PP wrote the manuscripts. RN and PP oversaw the completion of this study and finally edited the manuscript. All authors contributed to the article and approved the submitted version.

## Funding

This work was partially supported by IoE development fund, which was released for MG at the Institute of Medical Sciences, Banaras Hindu University. This submissions will utilize the pilot partnership between UNC Library and Frontiers (https://library.unc.edu/2022/10/frontiers-partnership/).

## Conflict of interest

The authors declare that the research was conducted in the absence of any commercial or financial relationships that could be construed as a potential conflict of interest.

## Publisher’s note

All claims expressed in this article are solely those of the authors and do not necessarily represent those of their affiliated organizations, or those of the publisher, the editors and the reviewers. Any product that may be evaluated in this article, or claim that may be made by its manufacturer, is not guaranteed or endorsed by the publisher.

## Abbreviations

PEI: polyethyleneimine
Ag-NPs: silver nanoparticles
PEI-f-Ag-NPs: polyethyleneimine-functionalized silver nanoparticles
MIC: minimum inhibitory concentration
BSA: bovine serum albumin
AgNO_3_: silver nitrate
μg/mL: microgram/milliliter
PB: Phosphate buffer
PBS: phosphate buffer saline
FL: Fluorescence
RPMI: Rosewell Park Memorial Institute Medium
YPD: Yeast Extract Peptone Dextrose.
